# Multicentric Reticulohistiocytosis mimicking seronegative rheumatoid arthritis: a case report of destructive erosive arthritis with partial response to Upadacitinib

**DOI:** 10.1093/omcr/omag174

**Published:** 2026-10-25

**Authors:** Muhayman Sadiq, Omar Ba-Abbad, James Galloway, Ernest Fonocho

**Affiliations:** McGovern Medical School at UTHealth Houston, Department of Diagnostic and Interventional Imaging, 6431 Fannin St, Houston, TX 77030, United States; King's College London, Guy’s, King’s and St Thomas’ School of Medicine, Guy’s Campus, Great Maze Pond, London SE1 1UL, United Kingdom; The London School of Economics and Political Science, Houghton Street, London WC2A 2AE, United Kingdom; King's College London, Guy’s, King’s and St Thomas’ School of Medicine, Guy’s Campus, Great Maze Pond, London SE1 1UL, United Kingdom; McGovern Medical School at UTHealth Houston, Department of Diagnostic and Interventional Imaging, 6431 Fannin St, Houston, TX 77030, United States

**Keywords:** multicentric reticulohistiocytosis, non-Langerhans cell histiocytosis, erosive arthritis, seronegative rheumatoid arthritis, Upadacitinib, JAK inhibition

## Abstract

Multicentric reticulohistiocytosis (MRH) is a rare non-Langerhans cell histiocytosis that can mimic seronegative rheumatoid arthritis through destructive inflammatory arthritis and papulonodular skin lesions. We report a 44-year-old woman with progressive polyarthritis, purplish papulonodular lesions and destructive erosive disease of the hands and feet. Rheumatoid factor, anti-cyclic citrullinated peptide antibody and antinuclear antibody were negative. Radiographs showed sharply demarcated erosions with prominent distal interphalangeal involvement, and skin biopsy confirmed MRH. Available malignancy screening, including oncology review, mammography, cervical screening and blood tests, was reassuring, although planned PET-CT was not completed due to loss to follow-up. Methotrexate and corticosteroids were not tolerated, certolizumab produced partial response, and upadacitinib led to modest cutaneous improvement but persistent active synovitis at four months. This case highlights MRH as a diagnostic mimic of seronegative rheumatoid arthritis and illustrates discordant treatment response to JAK inhibition.

## Introduction

Multicentric reticulohistiocytosis (MRH) is a rare non-Langerhans cell histiocytosis, with approximately 300–500 reported cases worldwide. It commonly presents with destructive inflammatory polyarthritis and papulonodular skin lesions and is associated with autoimmune disease and malignancy, making systemic evaluation important [1–3]. Radiographs may show well-demarcated marginal erosions involving the distal interphalangeal (DIP) joints, helping distinguish MRH from rheumatoid arthritis (RA), where DIP disease is less prominent [4]. Diagnosis relies on clinicoradiological correlation and biopsy, typically showing CD68-positive, S100/CD1a-negative histiocytes with eosinophilic ground-glass cytoplasm [1, 3]. We report biopsy-proven MRH mimicking seronegative RA and highlight discordant response to upadacitinib, with modest cutaneous improvement but persistent inflammatory arthritis.

## Case report

A 44-year-old woman with no known past medical history presented to the emergency department with a traumatic hand fracture, which prompted further evaluation. In the preceding month, she had developed progressive bilateral hand swelling, an erythematous macular rash on the arms and face, and purplish papulonodular lesions on the hands, nose and ears. Over the same period, she reported worsening inflammatory joint pain and morning stiffness affecting the knees, hands, wrists, elbows and hips, with progressive loss of hand function. She denied sensory disturbance. On examination, there was bilateral wrist swelling with reduced range of motion, flexion deformities of multiple distal and proximal interphalangeal joints, synovitis of the second and third metacarpophalangeal joints bilaterally, and bilateral knee effusions with severe synovitis.

Inflammatory markers were raised, with erythrocyte sedimentation rate 41 mm/h and C-reactive protein 22 mg/L. Rheumatoid factor, anti-cyclic citrullinated peptide antibody, antinuclear antibody and uric acid were negative or within normal range (Table 1).

**Table 1:** Summary of laboratory results at presentation. Abnormal values are shown in bold.

Parameter	Result	Reference Range
Haemoglobin (g/dL)	**11.0**	12–16
White Blood Cells (×10^9^/L)	**11.5**	4.0–10.0
Neutrophils (%)	**80**	40–75
Lymphocytes (%)	15	20–45
Platelets (×10^9^/L)	**403**	150–400
Erythrocyte Sedimentation Rate (mm/h)	**41**	<20
C-Reactive Protein (mg/L)	**22**	<5
Alanine Aminotransferase (U/L)	17	10–40
Aspartate Aminotransferase (U/L)	22	10–40
Alkaline Phosphatase (U/L)	76	44–147
Sodium (mmol/L)	138	135–145
Potassium (mmol/L)	4.1	3.5–5.1
Chloride (mmol/L)	102	98–107
Urea (mmol/L)	7	2.5–7.8
Creatinine (mg/dL)	0.9	0.6–1.1
eGFR (mL/min/1.73 m^2^)	110	>90
Rheumatoid Factor	Negative	—
Anti-CCP Antibody	Negative	—
Antinuclear Antibody (ANA)	Negative	—
Uric Acid (mg/dL)	4.4	3.4–7.0

Hand radiographs showed multifocal sharply demarcated erosions, most pronounced at the DIP joints, with involvement of the proximal interphalangeal, metacarpophalangeal, carpometacarpal, radiocarpal, ulnocarpal and intercarpal joints (Fig. 1). There was marked interphalangeal joint space narrowing, dorsal subluxation/dislocation of distal phalanges and swan-neck deformity. Foot radiographs showed bilateral articular and juxta-articular erosions involving the metatarsophalangeal and interphalangeal joints (Fig. 2). Skin biopsy from a dorsal finger nodule showed epithelioid histiocytes with dense eosinophilic cytoplasm, and immunohistochemistry was reported as supportive of MRH, consistent with non-Langerhans cell histiocytosis. However, original histopathology and immunohistochemistry images were not available for inclusion because the external provider did not release these materials to the authors.

**Figure 1:**
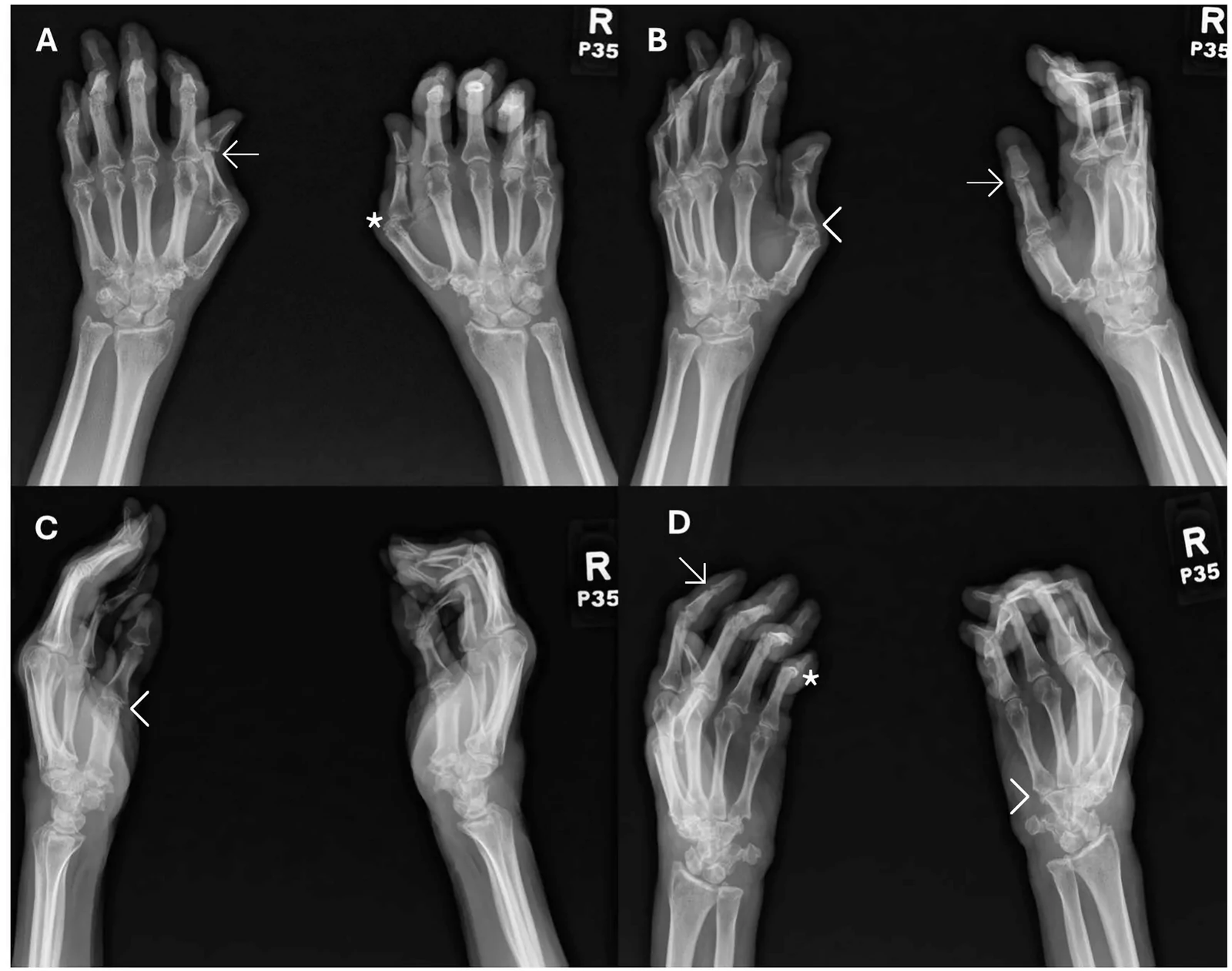
Hand radiographs in posteroanterior (A), oblique (B), lateral (C) and ball-catcher (D) views show diffuse, sharply demarcated erosive changes of the hand joints, most pronounced at the distal interphalangeal joints, compatible with MRH. Arrows indicate erosive changes of DIP joints. Arrowheads indicate erosive involvement at additional non-DIP joints. Asterisks indicate joint subluxation/deformity.

**Figure 2:**
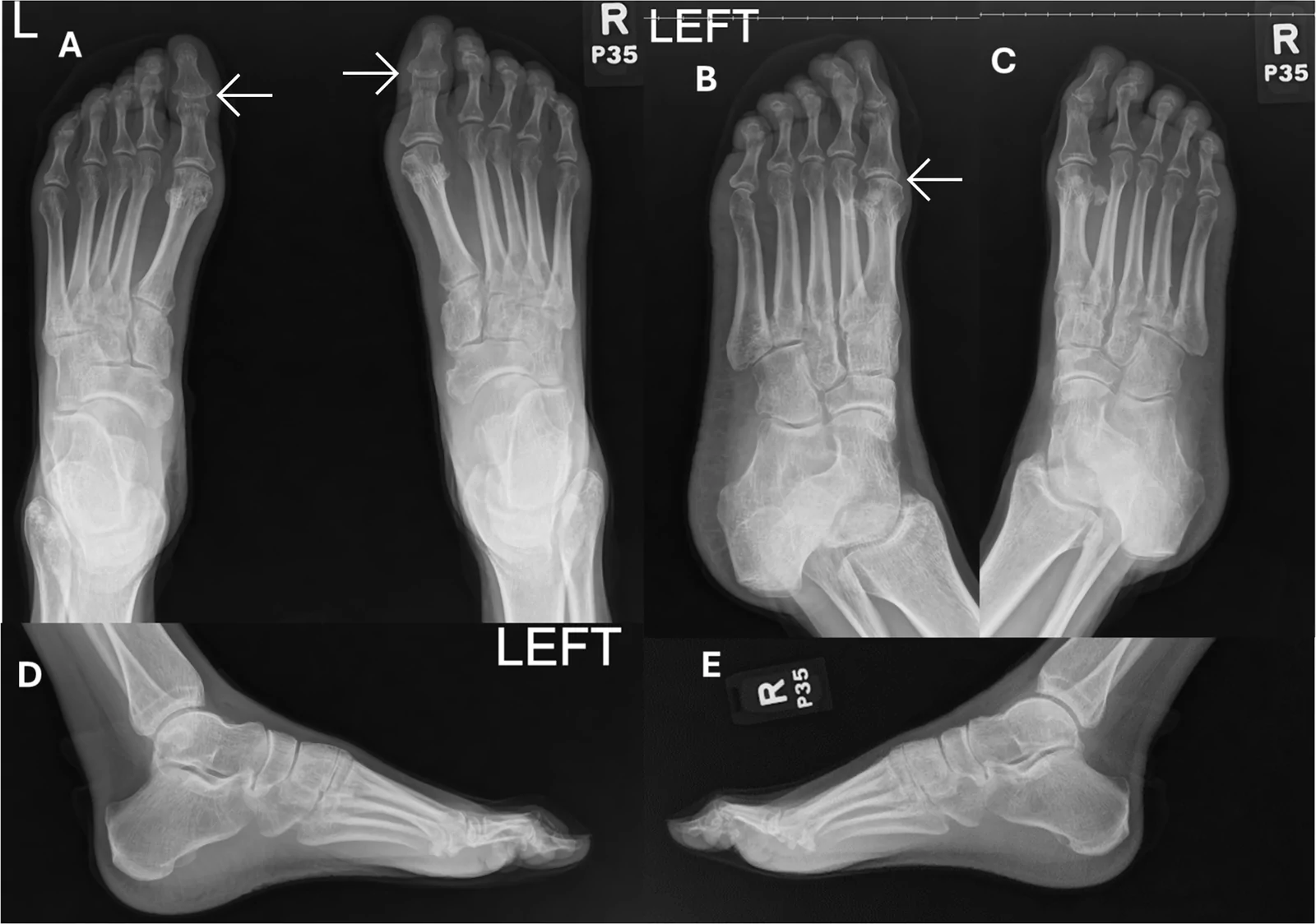
Foot radiographs in anteroposterior (A), oblique (B,C) and lateral (D,E) views showing bilateral multifocal articular and juxta-articular erosions at the metatarsophalangeal and interphalangeal joints, compatible with MRH (arrows).

Given the recognized malignancy association, systemic review and malignancy screening were considered. The patient reported intermittent fevers over the preceding two-year disease course but denied recent weight loss. She had previously been reviewed by oncology, and mammography, cervical screening and blood tests were reported as reassuring. She denied additional screening imaging. PET-CT was discussed to complete the systemic work-up and screen for occult malignancy but was not completed because follow-up was interrupted by difficulties obtaining PET-CT approval and reimbursement, together with payment-related barriers to further investigation.

Following diagnosis, methotrexate and oral corticosteroids were discontinued because of gastrointestinal intolerance. Certolizumab pegol was then started because tumour necrosis factor-alpha inhibitors have been reported to improve joint symptoms in MRH, but the initial partial response was not sustained [5]. Upadacitinib 15 mg once daily was therefore introduced for refractory disease, given limited reports of JAK inhibition in MRH and the biological plausibility of targeting cytokine-mediated inflammation [6, 7]. At four-month follow-up, she reported approximately 20% subjective cutaneous improvement with reduced nodule size and irritation, but persistent joint pain, swelling and synovitis affecting the hands, knees, elbows and feet. Inflammatory markers remained elevated, with ESR 50 mm/h and CRP 27 mg/L. Switching to infliximab was considered if disease activity worsened, and she was subsequently lost to follow-up.

## Discussion

This case highlights MRH as an uncommon but important mimic of seronegative RA. The combination of negative RF/anti-CCP/ANA serology, papulonodular lesions and DIP-predominant erosions prompted reconsideration of the initial differential diagnosis and biopsy rather than treatment escalation for presumed RA. The initial differential diagnosis included psoriatic arthritis, erosive osteoarthritis, gout and seronegative rheumatoid arthritis, all of which may involve the distal interphalangeal joints. However, the combination of papulonodular skin lesions, sharply demarcated erosions, negative serology and histopathological confirmation favored MRH over these alternatives. The distribution is clinically relevant: a single-centre comparison found DIP involvement in approximately 64% of MRH patients versus 24% of RA patients [4]. While radiographs are not diagnostic alone, sharply demarcated erosions, prominent DIP involvement and relative lack of classic periarticular osteopenia should raise suspicion when accompanied by characteristic skin lesions.

MRH has no standardised treatment pathway. Methotrexate and corticosteroids are commonly used, while biologics, cytotoxic agents and bisphosphonates have been reported with variable success [2, 3, 5]. Upadacitinib was selected because conventional therapy was not tolerated, TNF-alpha inhibition produced incomplete response, and JAK inhibition has been described in refractory MRH [6, 7].

In contrast to prior reports of marked improvement, our patient had modest cutaneous improvement and persistent inflammatory arthritis. The discordant response observed in our patient may reflect differences between inflammatory cutaneous disease and established erosive articular disease. One possible explanation is that cutaneous manifestations may be more responsive to cytokine modulation through JAK inhibition, whereas persistent articular disease may reflect osteoclast-mediated damage, a partly separate pathway that is less reversible once erosive change is established [8]. Accordingly, this case suggests that treatment response in MRH may be heterogeneous and that cutaneous and articular manifestations may not improve in parallel.

The incomplete malignancy evaluation is an important limitation. Although prior oncology review, mammography, cervical screening and blood tests were reassuring, planned PET-CT was not completed because of reimbursement and payment-related barriers, so occult malignancy cannot be definitively excluded. Follow-up was limited to four months after upadacitinib, preventing assessment of long-term response or radiographic progression. Additionally, although biopsy and immunohistochemistry were performed and supported MRH, the original histopathology and immunohistochemistry images were unavailable for inclusion because the external provider did not release these materials.

## Conclusion

This case highlights MRH as an important mimic of seronegative rheumatoid arthritis, particularly when destructive erosive arthritis involves the distal interphalangeal joints and is accompanied by papulonodular skin lesions. It also adds to the limited literature on JAK inhibition in MRH by illustrating a discordant response to upadacitinib, with modest cutaneous improvement but persistent inflammatory arthritis.
